# Early Obliterated Cabrol Shunt: Culprit of Aortopulmonary Fistula in Large Pseudoaneurysm after Bentall Procedure

**DOI:** 10.3390/jcdd9120449

**Published:** 2022-12-09

**Authors:** Bowen Zhang, Yanxiang Liu, Yaojun Dun, Xiaogang Sun

**Affiliations:** Department of Cardiovascular Surgery, Fuwai Hospital, National Center for Cardiovascular Diseases, Chinese Academy of Medical Sciences and Peking Union Medical College, Beijing 100037, China

**Keywords:** aortopulmonary fistula, pseudoaneurysm, Bentall, Cabrol shunt

## Abstract

Aortopulmonary fistula secondary to a large pseudoaneurysm after a Bentall procedure is a rare but complex complication. Herein, we report a case of Cabrol shunt obliteration and pseudoaneurysm formation three months after a Bentall procedure. The patient also presented with congestive heart failure due to an aortopulmonary fistula six years later. Surgery was successfully performed to repair the dehiscence of the biliteral coronary ostia and the aortopulmonary fistula, and to replace the ascending aorta. Postoperatively, the patient recovered uneventfully.

## 1. Introduction

Acquired aortopulmonary fistula is rare; however, it should be suspected in a patient who has a pseudoaneurysm after an ascending aorta procedure and presents with sudden onset of congestive heart failure [1,2,3]. Herein, we report an exceptional case of a 48-year-old man who had a large aortic pseudoaneurysm complicated by fistulization into the pulmonary artery six years after a Bentall procedure, which was successfully repaired with surgery.

## 2. Case Report

**Figure 1:**
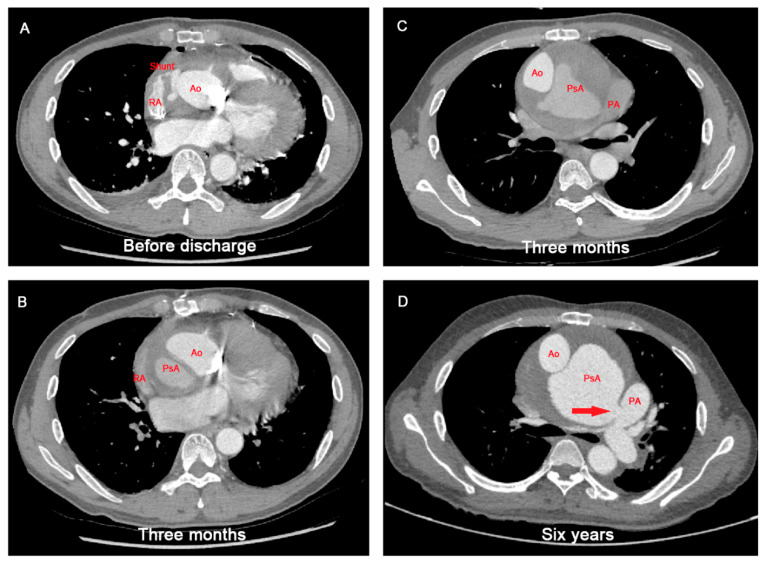
Aortic computed tomographic angiography scan showing the pathogenetic course of pseudoaneurysm and aortopulmonary fistula after Bentall procedure. (**A**) A patent Cabrol shunt after the initial Bentall procedure. (**B**,**C**) Cabrol shunt obliteration and pseudoaneurysm formation 3 months after the initial Bentall procedure. (**D**) Aortopulmonary fistula formation 6 years after the initial Bentall procedure. Ao: aorta; PA: pulmonary artery; PsA: pseudoaneurysm; RA: right atrium; red arrow indicates the aortopulmonary fistula.

Six years prior to presentation, this patient underwent a Bentall procedure using the “inclusion technique with Cabrol shunt (a shunt connecting the peri-graft space and the right atrium)” for an aortic root aneurysm. The etiology of the aneurysm was suspected to be atherosclerosis. Before discharge, aortic computed tomography angiography (CTA) indicated contrast filling in the peri-graft space and a patent Cabrol shunt (Figure 1A). An aortic CTA at three months showed dehiscence at the bilateral coronary ostia, Cabrol shunt obliteration and pseudoaneurysm formation (Figure 1B,C). The patient refused reoperation and underwent an annual aortic CTA scan at a local hospital. Six years later, he was readmitted to our institute with congestive heart failure, presenting as dyspnea and cough. On admission, physical examination revealed a systolic murmur (grade 3/6) at the second left intercostal space. His blood pressure was 110/70 mmHg, and had he had a sinus rhythm with normal conduction on an electrocardiogram. The aortic CTA revealed a large pseudoaneurysm (maximal diameter, 11.3 cm) fistulizing into the pulmonary artery and compressing the right pulmonary artery (Figure 1D). Transthoracic echocardiography revealed moderate tricuspid regurgitation and increased right ventricular systolic pressure (85 mmHg).

## 3. Surgical Repair

**Figure 2:**
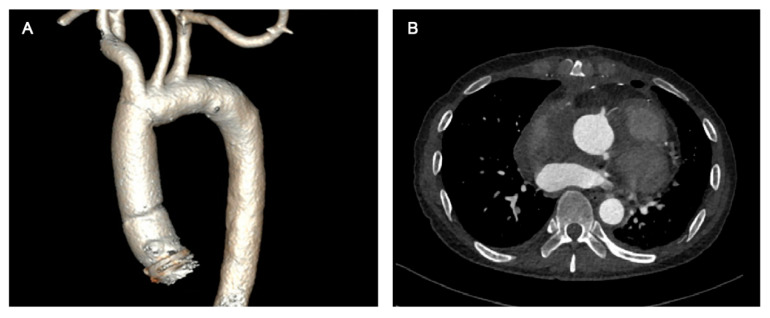
Postoperative aortic CTA scan revealed no leakage around the coronary ostia and an absence of pseudoaneurysm. (**A**) Three-dimensional reconstruction of aortic CTA; (**B**) Aortic CTA image at the coronary ostial level. CTA: computed tomographic angiography.

An occluded balloon was first deployed to the ascending aorta through the left femoral artery, which would act as a cross clamp to occlude the aorta if it was ruptured during sternotomy. Cardiopulmonary bypass (CPB) was established by the right femoral artery, right axillary artery and right femoral vein before sternotomy, but was not instituted during sternotomy. After sternotomy, the CPB was instituted and the aorta was cross-clamped between the innominate artery (IA) and left common carotid artery (CCA) after lysis of the mediastinum. The IA was clamped and the right CCA was perfused via the right axillary cannulation. Then, the ascending aorta was incised and histidine-tryptophan-ketoglutarate solution (HTK) solution was perfused anteriorly via the coronary ostia. Dehiscence at the bilateral coronary ostia was interrupted sutured by 5/0 polypropylene sutures. The fistula on the pulmonary artery trunk was running sutured by 5/0 polypropylene sutures. Once a core temperature of 24 °C was reached, deep hypothermic circulatory arrest (DHCA) and bilateral antegrade cerebral perfusion was initiated. The original distal anastomosis was resected and the ascending aorta was replaced using a new 28-mm vascular prosthesis with distal anastomosis performed under DHCA. When the distal anastomosis was completed, perfusion of the lower body was resumed. Proximal ascending aorta anastomosis, rewarming and closure completed the operation. The surgery was performed successfully, and the patient recovered uneventfully. His aortic CTA before discharge showed no leakage around the coronary ostia and an absence of pseudoaneurysm (Figure 2).

## 4. Discussion

Aortopulmonary fistula secondary to a large pseudoaneurysm after a Bentall procedure is rare, but complex. Despite successful surgical treatment and uneventful recovery, some experience and lessons are worth summarizing.

The “Inclusion technique,” in the Bentall procedure, has the advantages of avoiding extensive dissociation of the aortic root and simplifying hemostasis compared with the “button technique,” but it has a higher probability of causing pseudoaneurysm formation. An appropriate “Cabrol shunt” reduces the pressure on the peri-graft space and avoids the dehiscence of coronary anastomosis as well as pseudoaneurysm formation [4]. Therefore, the “inclusion technique with Cabrol shunt” is currently mainstream in our institute. Generally, the peri-graft space and the Cabrol shunt were obliterated successively several months postoperatively. Unfortunately, this is a failure case of using the “inclusion technique with Cabrol shunt.” For this case, the pseudoaneurysm might begin with minor leakage of the coronary artery anastomosis. When the “Cabrol shunt” is patent, the pressure of peri-graft space is low and the closure of minor coronary artery leakage is possible. Unfortunately, the “Cabrol shunt” which was obliterated early further increased the pressure on the peri-graft space, which in turns exacerbated dehiscence of anastomosis and pseudoaneurysm dilation. Eventually, the pseudoaneurysm dilated severely, compressed the right pulmonary artery and finally fistulized into the pulmonary artery trunk.

DHCA was reported to avoid pseudoaneurysm rupture during sternotomy, and balloon occlusion of the ascending aorta distal to the aortopulmonary fistula was reported to reduce aortopulmonary shunting from the CPB before the aorta was cross-clamped [2,3]. These methods required the placement of a left ventricular vent in advance via the right thoracic, as well as prolonged CPB or DHCA time. To ensure safety, CPB and an occlusion balloon were prepared for this case, but we did not use them during the sternotomy, as we considered that the pseudoaneurysm was less likely to rupture with careful operation. In addition, the aorta was clamped on the aortic arch between the IA and left CCA, thus avoiding extensive dissociation of the pseudoaneurysm under perfusion, and the aorta was clamped distal to the aortopulmonary fistula in order to cease the aortopulmonary shunting.

## 5. Conclusions

In summary, aortopulmonary fistula secondary to a large pseudoaneurysm after a Bentall procedure is a rare, but complex, complication, which is sometimes attributed to the inappropriate use of the “inclusion with Cabrol shunt” technique during the Bentall procedure. With the application of the “inclusion with Cabrol shunt” technique in the Bentall procedure, full-thickness sutures, careful scrutiny for hemorrhage and an appropriate-sized Cabrol shunt play crucial roles in avoiding the formation of large pseudoaneurysms which are complicated by fistulization into the pulmonary artery.

## Data Availability

Not applicable.
